# Notes and Notices

**Published:** 1883-09

**Authors:** 


					﻿NOTES AND NOTICES.
Homceopathic Medical Society of New Yoke meets at Ithaca, Sep-
tember 11th and 12th. Semi-annual meeting.
The Pennsylvania State Society meets in Philadelphia, September
18th and 20th. Annual meeting.
Quack Remedies.—After a long run of a patent medicine as a cure for
lung troubles, a new run may be established by calling it a remedy for stom-
ach troubles. When a fortune has been made out of lung pads, they can be-
cut down in size and another fortune made out of them as kidney pads.
				

